# Inactivation of the *porB* gene reduces the virulence of *Neisseria meningitidis* in transgenic mice

**DOI:** 10.1186/s12866-025-04246-3

**Published:** 2025-08-16

**Authors:** Cecilia Klanger, Ala-Eddine Deghmane, Lorraine Eriksson, Olof Säll, Sara Thulin Hedberg, Paula Mölling, Muhamed-Kheir Taha

**Affiliations:** 1 Department of Laboratory Medicine, Faculty of Medicine and Health, Örebro University, Örebro, Sweden; 2 Invasive Infections Unit, Institut Pasteur, Paris, France; 3 Department of Infectious Diseases, Faculty of Medicine and Health, Örebro University, Örebro, Sweden

**Keywords:** *Neisseria meningitidis*, Carriage, Invasive meningococcal disease, Transgenic mice, *PorB*, Inflammatory cytokines, Experimental infection

## Abstract

**Background:**

*Neisseria meningitidis* is a human pathogen, carried asymptomatically in the nasopharynx, that can also cause invasive meningococcal disease. Understanding the carriage/invasiveness balance is crucial, and bacterial genetic factors may impact this balance. A previous genome-wide association study reported that the gene *porB* class 3 was significantly associated with carriage isolates. This study aimed to examine the impact of *porB* variants on virulence in carriage and invasive meningococcal isolates.

**Results:**

For this, 24 isolates were used (13 invasive and 11 carriage) belonging to different genogroups (B, C, W, Y, and cnl) and selected based on the presence of the genetic variant *porB* class 2 or class 3. Transgenic BALB/c mice expressing human transferrin were infected intraperitoneally with these isolates. After 3 and 24 h of infection, clinical scores (fur quality, strength, and temperature) and bacterial load in blood were used to evaluate bacterial virulence. The concentrations of inflammatory cytokines were determined from blood. PorB^-^ mutants were created from a carriage and an invasive isolate, and were tested in transgenic mice. The invasive isolates provoked significantly more severe infections compared to the carriage isolates, and the carriage isolates of *porB* class 3 were significantly less virulent than the invasive isolates of *porB* class 2 or 3. The invasive PorB^-^ isolate caused milder infections than the parental isolate.

**Conclusions:**

This study confirms the ability of invasive isolates of *N. meningitidis* to cause more severe infections than carriage isolates in transgenic mice. The *porB* expressed in invasive isolates seems to contribute to their higher virulence compared to carriage isolates, although this effect may depend on the genomic context. Notably, differences in virulence were mainly observed among serogroup C and W isolates.

**Supplementary Information:**

The online version contains supplementary material available at 10.1186/s12866-025-04246-3.

## Background

*Neisseria meningitidis*, also known as meningococcus, is a human pathogen that colonises the nasopharynx and can cause invasive meningococcal disease (IMD) including septicaemia and meningitis [1, 2]. IMD is rare, but has a high burden of disease. It is still unclear how bacteria and host interaction can cause a switch from asymptomatic carriage to IMD. The incidence of IMD varies widely across the world and by age. Fatality rates range from 2 to 20% with correct treatment, but can reach 80% in untreated cases. Up to 25% of survivors are left with lifelong disabling sequelae [3, 4]. Meningococcal carriage rates vary by region and age, with the highest rates (10–35%) seen among young adults [5, 6]. Among twelve defined serogroups, six major serogroups (A, B, C, Y, W, and X) are the leading cause of meningococcal disease globally. Non-capsulated isolates, known as capsule null locus (cnl), are generally less invasive among immunocompetent individuals but are common in carriers [2]. Meningococci can also be classified by multilocus sequence typing into sequence types (ST) and clonal complexes (cc) [7], with cc11, cc32, cc269, and cc41/44 being hyperinvasive and over-represented in invasive cases [5, 8, 9].

The pathogenesis of IMD results from an interaction of host factors and susceptibility, environmental factors such as exposure to the wind-driven dust of the Harmattan, and microbial factors [2, 10]. Host factors include complement component deficiencies or treatment with complement inhibitors, asplenia, HIV infection, other types of immunosuppression, and smoking [11, 12]. Microbial virulence factors include the polysaccharide capsule, the outer membrane proteins (OMPs, e.g. PorA and PorB), adhesion molecules (pili and opacity proteins; Opa and Opc), the iron acquisition mechanisms, and the endotoxin lipooligosaccharide (LOS) [2, 13]. Although several virulence factors of *N. meningitidis* are known, with the most important being the capsule expression [14], it is largely unknown which genetic elements are associated with invasive disease [5], and thus why some isolates lead to carriage while others cause severe infection.

In our previous genome-wide association study (GWAS), we analysed carriage and invasive *N. meningitidis* isolates [15]. The study identified single nucleotide polymorphisms (SNPs) and genes associated with isolates of either carriage or invasive type. Among these genes was *porB* class 3, associated with carriage isolates. The *porB* gene encodes for porin B (PorB), a major OMP. This voltage-gated porin is a known virulence factor for *Neisseria* species, and is involved in host-cell interaction [2, 16]. In any given strain of *N. meningitidis*, PorB is encoded by one of two homolog families, to which the allele of the *porB* locus is assigned: *porB* class 2 and *porB* class 3 [17].

In the present study, we built on our previous findings by using an animal model with transgenic BALB/c mice expressing human transferrin [18, 19]. The aim was to examine the virulence of carriage and invasive isolates of *N. meningitidis*, focusing on genetic variants of *porB* and isolates with inactivated *porB*, in humanized mice.

## Methods

### Bacterial isolates

Bacterial isolates used in this study were chosen from the earlier GWAS study (Table 1) [15]. The carriage isolates (*n* = 11) were collected during a carriage study among Swedish university students [20], and the invasive isolates (*n* = 13) were collected in Sweden during the same years, 2018 and 2019. These 24 isolates belonged to different genogroups (B, C, W, Y, and cnl) and were selected to ensure overall genetic similarity between the isolates, with the primary difference being the presence of either *porB* class 2 or class 3. Capsule expression of the isolates was determined using co-agglutination (Difco antisera, BD Diagnostics), and was categorised as positive (+), negative (-), or, if the result was uncertain, as positive/negative (+/-). The strength of the agglutination reaction was assessed qualitatively, and isolates marked as +/– showed weaker reactions compared to strongly positive (+) isolates, but not absent reactions as in the negative isolates. Isolates were grown on gonococcal agar plates (3.6% Difco GC Medium Base agar, BD Diagnostics) before co-agglutination, and on agar plates containing GCB medium (Difco, Thermo Fisher Scientific) supplemented with Kellogg’s supplements I and II [21] for the other experiments. The isolates were incubated on the agar plates overnight, for 18–20 h, in a humid atmosphere with 5% CO_2_ at 37° C.

**Table 1:** Basic characteristics of* Neisseria meningitidis *isolates included in this study (n = 26). ST = sequence type; cnl = capsule null locus

Isolate	Genogroup	Clinical characteristic	Clonal complex	*porB* class	Capsule expression ^a^	Origin
19 − 3	Y	Invasive	ST-23	2	+	[15]
19–192	Y	Invasive	ST-23	2	+	[15]
McBar-1234	Y	Carriage	ST-23	3	+	[20]
McBar-1425	Y	Carriage	ST-23	3	-	[20]
19–926	Y	Invasive	ST-23	3	+	[15]
19–978	Y	Invasive	ST-23	3	+	[15]
McBar-21	Y	Carriage	ST-23	2	+	[20]
18–122	W	Invasive	ST-11	2	+	[15]
18–391	W	Invasive	ST-11	2	+	[15]
18–62	W	Invasive	ST-11	2	+	[15]
McBar-1399	W	Carriage	ST-11	2	+/-	[20]
19–419	C	Invasive	ST-32	3	+	[15]
19–977	C	Invasive	ST-32	3	+	[15]
McBar-1859	C	Carriage	ST-32	3	-	[20]
McBar-2341	C	Carriage	ST-32	3	+	[20]
McBar-621	B	Carriage	ST-32	3	-	[20]
19–853	B	Invasive	ST-32	2	+	[15]
19–30	B	Invasive	ST-41/44	3	+/-	[15]
McBar-1351	B	Carriage	ST-41/44	3	-	[20]
McBar-1820	B	Carriage	ST-41/44	3	-	[20]
McBar-1226	cnl	Carriage	ST-41/44	3	Not applicable	[20]
18–641	cnl	Invasive	ST-41/44	3	Not applicable	[15]
McBar-1322	B	Carriage	ST-35	3	+	[20]
19-708-1	B	Invasive	ST-35	3	+	[15]
CK5 ^b^	Y	Carriage	ST-23	Not applicable	+	This study
CK8^b^	C	Invasive	ST-32	Not applicable	+	This study

^a^ Level of capsule as determined by co-agglutination, expressed as positive (+), negative (-), or uncertain (+/-)

b *por*B mutant harbouring an inactivated *por*B gene. CK 5 was derived from McBar-21,and CK8 was derived from 19 to 977

### Construction of *porB* mutants

Isolates were selected for creation of *porB* mutants based on the carriage and invasive potential observed in mice and the demonstrated capsule expression: the carriage isolate McBar-21 (mutant CK5) and the invasive isolate 19–977 (mutant CK8) (Table 1). The *porB* mutants were created by transformation of the previously described plasmid pGEM-porB::erm [22]. Transformants were selected on GCB agar plates supplemented with erythromycin at 2 µg/mL. Inactivation of *porB* was verified by PCR, using primers porB1F and porB100R and ERAM-1 and ERAM-3 (Supplementary Material Table S1) [22], and analysed by gel electrophoresis.

### Experimental infection in mice

The congenic BALB/c transgenic mice expressing human transferrin used in this study were established in the laboratory at the Institut Pasteur (using BALB/c mice from Janvier Labs, France) as previously described [19]. The mice were bred in-house and kept in a biosafety containment facility in filter-topped cages with sterile litter, water, and food, according to institutional guidelines. Female mice aged 8–12 weeks were used. The mice were injected intraperitoneally with 0.5 mL bacterial suspension of 5 × 10^7^ colony-forming units (CFU)/mL. Since the injection was considered a low-pain procedure, no anaesthesia was used. The carriage (*n* = 11) and invasive (*n* = 13) *N. meningitidis* isolates (Table 1) were tested in mice to assess their effects on clinical and biological markers of infection. Each mouse was injected with bacterial suspension of one isolate. All isolates were run in duplicate, using two separate experiments. NaCl (0.9%) was injected as negative control. Additional infection experiments were also performed for two of the isolates, one carrier and one invasive isolate, and their respective *porB* mutants (Table 1). The carriage isolate and its mutant were tested in two separate experiments with 3 and 5 mice per isolate, respectively (8 mice in total per isolate). The invasive isolate and its mutant were tested in three separate experiments with 3, 6, and 6 mice per isolate, respectively (15 mice in total per isolate). For all experiments, a total of 100 mice were used.

To determine the severity of infection with *N. meningitidis* in mice, the clinical outcomes of infection were assessed before infection (0 h) and at 3 and at 24 h post-infection, by scoring the quality of the fur, the strength, and the temperature of the mouse. Clinical observations were performed by the same assessor using standardised criteria and were not blinded to isolate type. Efforts were made to minimise potential bias through consistent evaluation. Hypothermia has been noted as a symptom of severe meningococcal infection in mice [23]. Fur quality was scored from 0 to 3, with 0 indicating erected and ruffled fur and 3 indicating glossy and smooth fur. Strength was scored from 0 to 5 by measuring the number of metallic chains a mouse could lift. Transcutaneous temperature was measured using an infrared thermometer for rodents (Bioseb, Vitrolles, France). The initial bacterial inoculum was determined, as well as bacterial loads (CFU/mL) in blood. Blood was drawn retro-orbitally at 3 h and 24 h post-infection. Serial dilutions were plated onto GCB plates that were incubated overnight at 37 °C in 5% CO_2_, and the bacterial count was then determined.

The cytokines interleukin-6 (IL-6), tumour necrosis factor α (TNF-α), and CXC chemokine ligand 1/keratinocyte-derived cytokine (CXCL1/KC) in sera from the 3 h and 24 h blood draws were measured using enzyme-linked immunosorbent assay (ELISA). Samples of sera were diluted 10 to 1 000 times, depending on the immunoassays for different cytokines. Kits for IL-6, TNF-α, and CXCL1/KC (R&D Systems) were used according to their respective protocols.

Animals were euthanised by cervical dislocation, in accordance with institutional and national ethical guidelines, and in compliance with the AVMA Guidelines for the Euthanasia of Animals (2020) [24]. The procedure was performed by personnel with demonstrated high technical proficiency and was selected for its ability to ensure rapid, humane death in small rodents. When necessary, topical ophthalmic anaesthetic (proparacaine 0.5% with heparin 5 U/mL) was applied. All efforts were made to ensure the animals were treated with care and euthanised as humanely as possible.

### Statistical measures

Clinical infection outcomes were expressed as medians. The Mann-Whitney U-test was used to compare the outcome of infection between mice infected with the carriage and invasive isolates, between isolates of *porB* class 2 and 3, and between the parental isolates with their respective mutants. The data for IL-6 and CXCL1 were log2 transformed after adding an arbitrary value of 1 to all samples. A *p*-value of < 0.05 was considered significant. All statistics were calculated using version 10.4.0 of GraphPad Prism (GraphPad Software).

## Results

### Evaluation of infection outcomes in mice

Data on the co-agglutination tests for the capsule expression of the 24 bacterial isolates are presented in Table 1. Of the 24 isolates, 15 were positive by co-agglutination, five were negative (all carriage isolates), and two were uncertain. Two isolates were cnl.

**Fig. 1:**
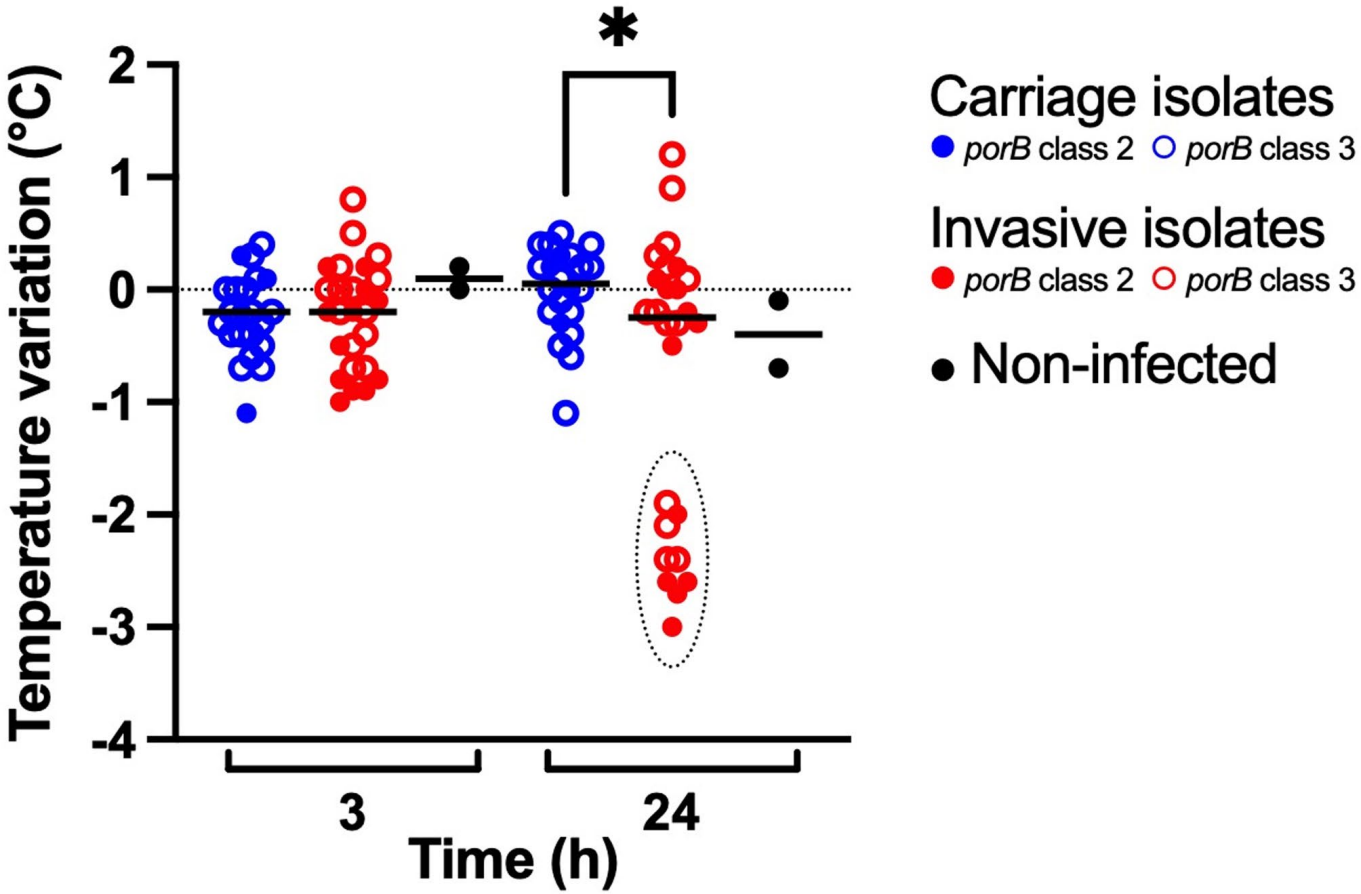
Temperature variation in mice infected with carriage and invasive isolates of *Neisseria meningitidis*, at 3 h and 24 h post-infection from each experiment. Non-infected mice are also included. The bars represent medians. Statistical significance was calculated using the Mann-Whitney U-test. At 24 h,the mice infected with invasive isolates showed significantly lower temperature than the mice infected with carriage isolates (*p* < 0.05). The dotted oval at 24 h highlights isolates of serogroups C and W. Data on capsule expression are shown in Table 1

**Fig. 2:**
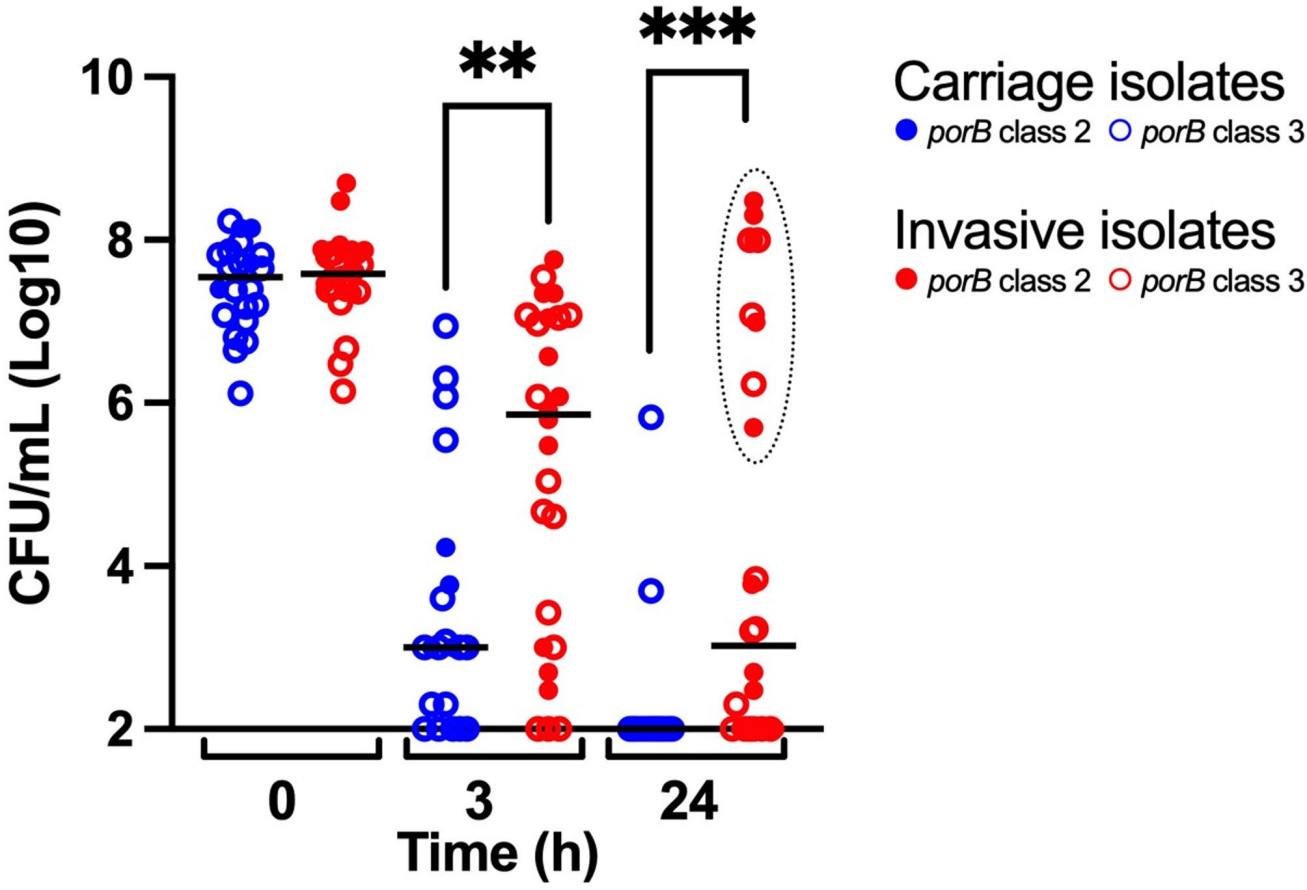
Bacterial load in mice infected with carriage and invasive isolates of *Neisseria meningitidis* at the time of infection (0 h; representing the inoculum) and at 3 h and 24 h post-infection from each experiment. The bacterial load at 0 h reflects the initial concentration of the bacterial suspension used for intraperitoneal injection and not the bacterial load in blood. Bars represent medians. Statistical significance was calculated using the Mann-Whitney U-test. The mice infected with invasive isolates showed a significantly higher number of bacteria than the mice infected with carriage isolates at both 3 h (*p* < 0.01) and 24 h (*p* < 0.001). The dotted oval at 24 h highlights isolates of serogroups C and W. Data on capsule expression are shown in Table 1

**Fig. 3:**
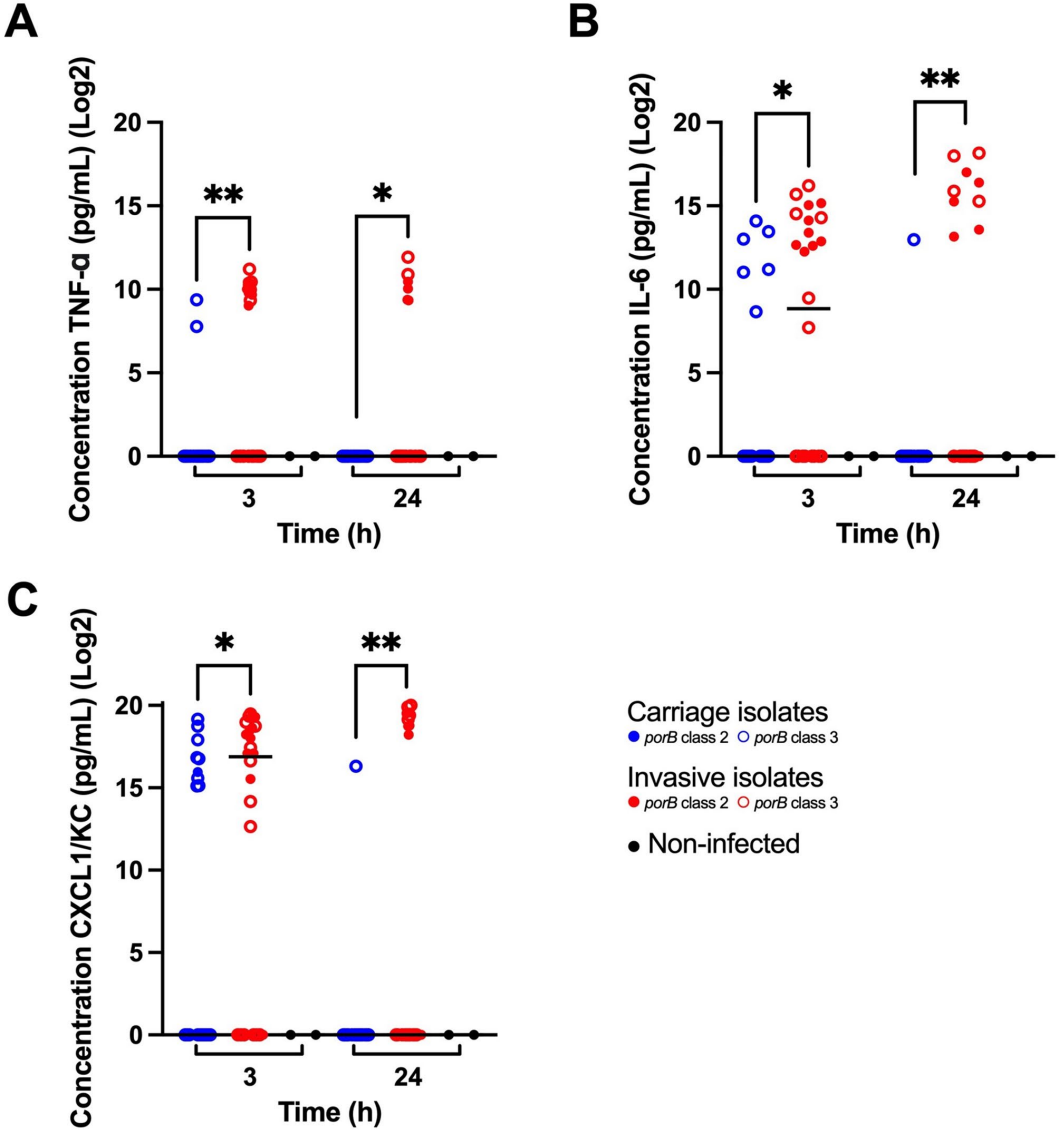
Concentrations of cytokines 3 h and 24 h post-infection in mice infected with carriage and invasive isolates of *Neisseria meningitidis*, and in non-infected mice from each experiment. Results are expressed as medians. Statistical significance was calculated using the Mann-Whitney U-test. (**A**) TNF-α levels were significantly higher in the mice infected with invasive isolates at 3 h (*p* < 0.01) and 24 h (*p *< 0.05). (**B**) IL-6 levels were significantly higher in the mice infected with the invasive isolates at 3 h (*p* < 0.05) and 24 h (*p* < 0.01). (**C**) CXCL1/KC levels were significantly higher in the mice infected with the invasive isolates at 3 h (*p* < 0.05) and 24 h (*p* < 0.01)

All 24 isolates were tested in transgenic mice to assess their effects on clinical and biological markers of infection. After infection with the original isolates, the mice infected with carriage versus invasive isolates showed significant differences in clinical outcomes, bacterial loads, and cytokine levels. Mice infected with invasive isolates were more ill at 24 h post-infection, as indicated by significantly lower temperature (Fig. 1) and scores for fur and strength (Supplementary Material Figure S1). This observation was corroborated by significantly higher bacterial loads in blood from mice infected with invasive isolates compared to carriage isolates (Fig. 2) at 3 h and 24 h post-infection. Post-infection cytokine levels were also significantly higher in mice infected with invasive isolates. For TNF-α, the difference was more significant at 3 h post-infection (Fig. 3A). For IL-6 (Fig. 3B) and CXCL1/KC (Fig. 3C), the difference was more significant at 24 h post-infection. The variation of all scores within the groups was greater in the mice infected with invasive isolates, compared to mice infected with the carriage isolates.

Further analyses showed no significant differences in the outcomes of infection between *porB* class 2 or 3, or between invasive isolates of different *porB* class (Figs. 1, 2 and 3). Carriage isolates of *porB* class 3 were significantly less virulent than both invasive isolates of *porB* class 3 at 24 h (*p* 0.0043) and invasive isolates of *porB* class 2 at 3 h (*p* 0.0058) and 24 h (*p* 0.0010). However, carriage isolates of *porB* class 2 did not show lower virulence than invasive isolates of either *porB* class 2 or *porB* class 3.

Two groups were identified among the mice infected with invasive isolates: one infected with isolates that showed high virulence, and one infected with isolates that showed lower virulence. This was most evident at 24 h post-infection. The isolates that showed high virulence all belonged to serogroups C and W (Figs. 1, 2 and 3) but were of different *porB* class (Table 1).

### Infection in mice with *porB* mutants

**Fig. 4:**
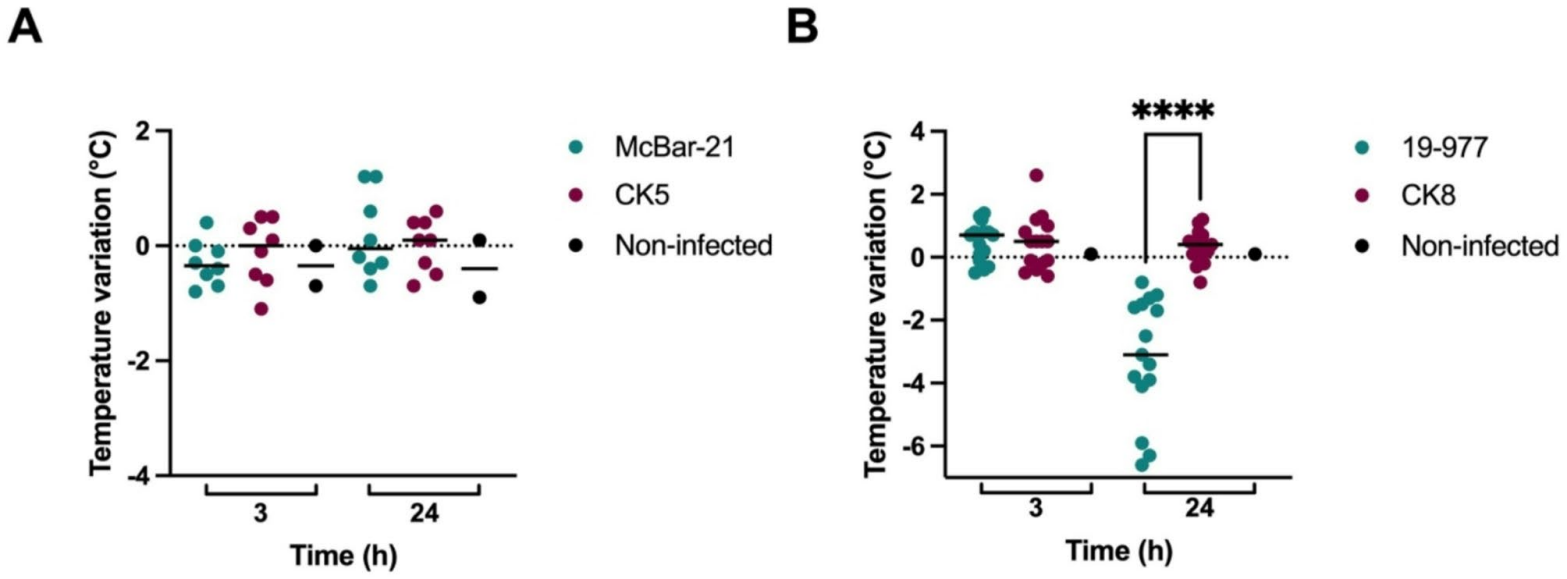
Temperature variation in mice infected with the parental isolates McBar-21 (carriage) or 19–977 (invasive) and their respective *porB* mutants CK5 and CK8,at 3 h and 24 h post-infection. Bars represent medians. Statistical significance was calculated using the Mann-Whitney U-test. Non-infected mice are also included. (**A**) Mice infected with the parental carriage isolate McBar-21 and those infected with its *porB* mutant CK5 showed no significant differences. (**B**) Mice infected with the parental invasive isolate 19–977 showed a significantly lower temperature than those infected with its *porB*mutant CK8 at 24 h (*p* < 0.0001)

**Fig. 5:**
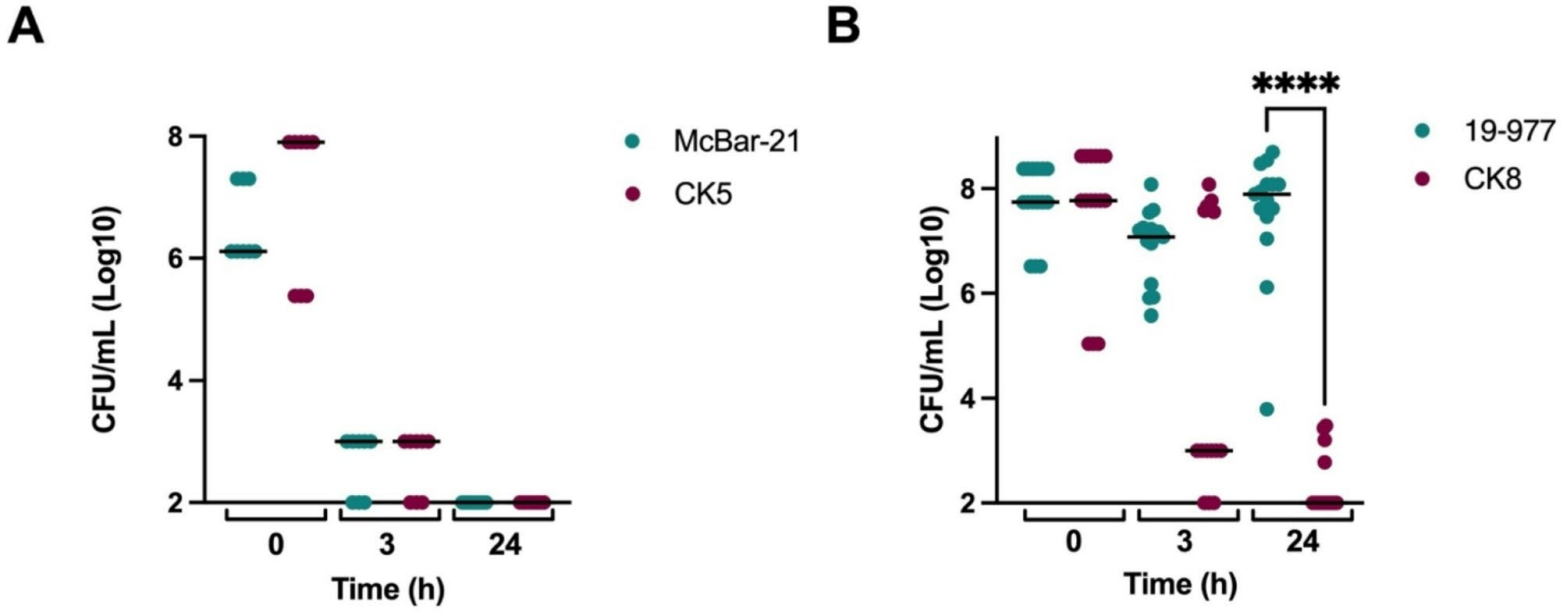
Bacterial load in mice infected with the parental isolates McBar-21 (carriage) or 19–977 (invasive) and their respective *porB*mutants CK5 and CK8, at the time of infection (0 h; representing the inoculum) and at 3 h and 24 h post-infection. The bacterial load at 0 h reflects the initial concentration of the bacterial suspension used for intraperitoneal injection and not the bacterial load in blood. Bars represent medians. Statistical significance was calculated using the Mann-Whitney U-test. (**A**) Mice infected with the parental carriage isolate Mcbar-21 and those infected with its *porB* mutant CK5 showed no significant differences. (**B**) Mice infected with the parental invasive isolate 19–977 showed a significantly higher number of bacteria than those infected with its *porB* mutant CK8 at 24 h (*p* < 0.0001).

**Fig. 6:**
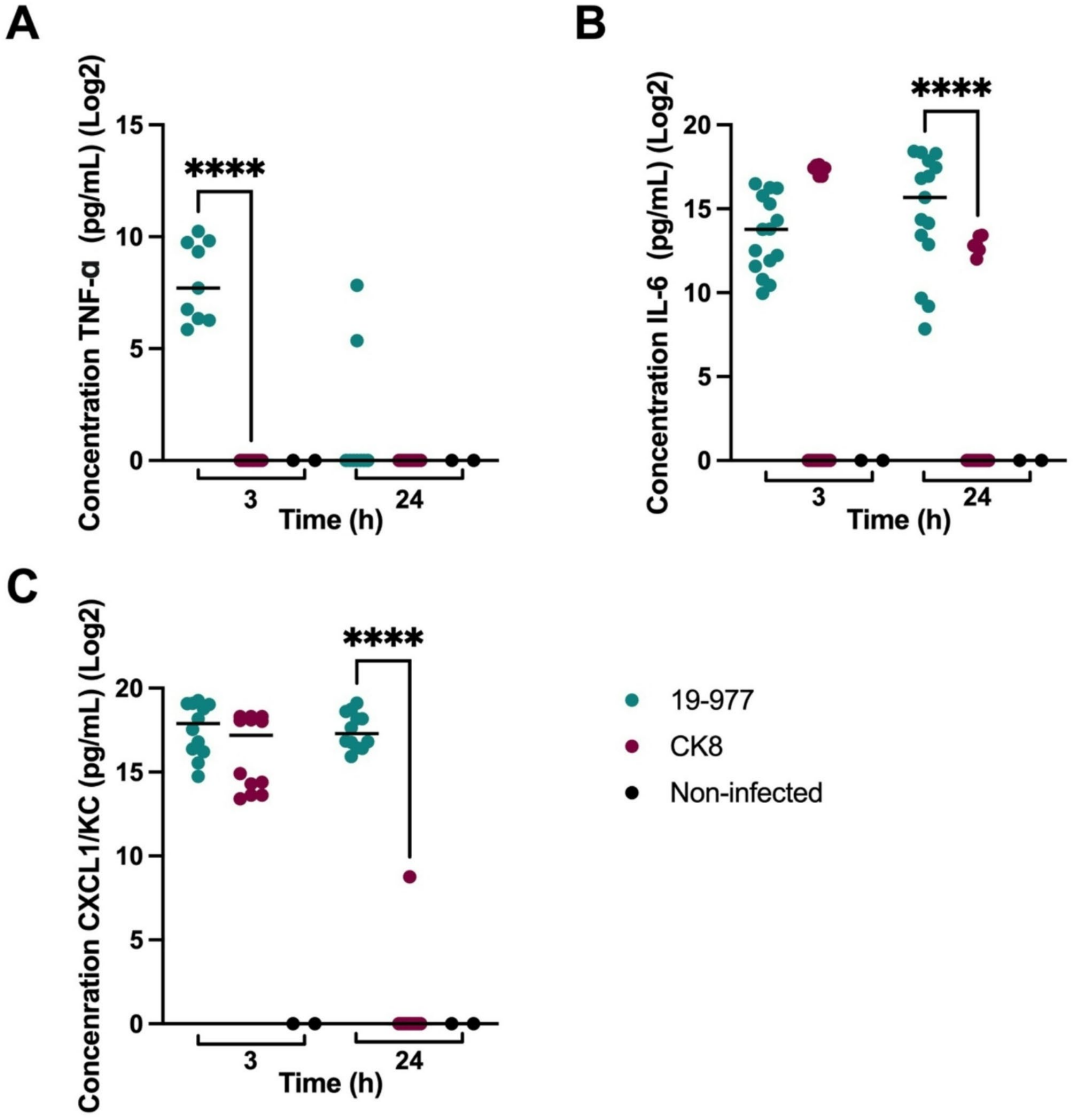
Concentrations of cytokines 3 h and 24 h post-infection in mice infected with the parental invasive isolate 19–977 and its *porB* mutant CK8 and in non-infected mice from each experiment. Bars represent medians. Statistical significance was calculated using the Mann-Whitney U-test. (**A**) TNF-α levels were significantly higher in the mice infected with the parental isolate at 3 h (*p* < 0.0001), with no significant difference at 24 h. (**B**) IL-6 levels were significantly higher in the mice infected with the parental isolate at 24 h (*p* < 0.0001),with no significant difference at 3 h. (**C**) CXCL1/KC levels were significantly higher in the mice infected with the parental isolate at 24 h (*p* < 0.0001), with no significant difference at 3 h

Mice infected with the carriage isolate McBar-21 and its mutant CK5 (harbouring an inactivated *porB* gene) showed no significant differences in post-infection clinical scores (Fig. 4A and Supplementary Material Figure S2). Mice infected with the invasive isolate 19–977 had a significantly lower temperature 24 h post-infection in comparison to those infected with its mutant CK8 (Fig. 4B). Other clinical markers such as fur quality and strength reflected the same pattern (Supplementary Material Figure S2). Moreover, the mice infected with 19–977 showed a significantly higher bacterial load than their counterparts infected with CK8 (Fig. 5B). The mice infected with this invasive mutant, harbouring an inactivated *porB* gene, seem to have cleared the bacteria to a similar extent as the mice infected with the carriage isolates (Fig. 5A). The cytokine levels followed the same pattern, being significantly higher in the mice infected with 19–977 than in those infected with CK8 (Fig. 6). For TNF-α, the difference was more significant at 3 h post-infection (Fig. 6B), while for IL-6 (Fig. 6B) and CXC1/KC (Fig. 6C), the difference was more significant at 24 h post-infection.

## Discussion

The use of this mouse model allowed a comparison of the virulence of invasive and carriage isolates of *N. meningitidis*. The invasive isolates were more virulent in mice, emphasizing intrinsic properties of the bacteria. The mouse model provided both clinical scores and biological scores using bacterial loads and inflammatory cytokines in the blood. There were some differences regarding *porB* class in these isolates; carriage isolates of *porB* class 3 were less virulent than invasive isolates of either class 2 or 3, but carriage isolates of *porB* class 2 showed no such difference. The invasive isolate that was mutated with inactivated *porB* was less virulent than its parental isolate, highlighting the importance of *porB* as a virulence factor.

The mice infected with the carriage isolates generally had a milder infection than the mice infected with invasive isolates, as revealed by several parameters. For instance, those with the invasive isolates had a significantly lower temperature at 24 h. This reflects a more severe infection in these mice, as hypothermia may be a result of severe meningococcal disease in mice [23]. Cytokine levels are generally correlated to the severity of IMD and fatality. Among these, the pro-inflammatory cytokines IL-6, which stimulates acute phase responses, haematopoiesis, and immune reactions [25], and TNF-α, a central early immune response activator [26], have both been related to meningococcal disease severity [27, 28]. The chemokine CXCL1/KC acts by recruiting and activating neutrophils as a host immune response to infection [29, 30], and has been linked to meningococcal infection in this mouse model [23]. TNF-α being an early immune response activator may explain why the difference was more significant at 3 h post-infection than at 24 h in all experiments where a rise in TNF-α levels was observed.

No differences in virulence were found when comparing isolates of *porB* class 2 with isolates *porB* class 3 on their own. However, when comparing carriage isolates of *porB* class 3 with invasive isolates of *porB* class 2, the carriage isolates caused significantly lower level of bacteriaemia at both 3 and 24 h, and when comparing them to invasive isolates of *porB* class 3, they were also significantly less virulent at 24 h. Such a difference was not observed when comparing carriage isolates of class 2 with invasive isolates of either *porB* class. These results are in line with the previous GWAS, which found a significant association between carriage isolates and *porB* class 3 [15].

The mouse model also made it possible to examine differences between invasive isolates, revealing that not all of the invasive isolates were equally virulent. There seemed to be two groups: one group of highly invasive serogroup C (ST-32 clonal complex) and serogroup W (ST-11 clonal complex) isolates, and one group of less invasive isolates. This points to the role of serogroups and clonal complexes. The evidently more invasive isolates in this study were exclusively of serogroups C and W, while the less invasive isolates were mainly of other serogroups (B, Y, cnl). Similarly, a previous study comparing differences in virulence between serogroups Y and W found that in mice expressing human transferrin, meningococci of serogroup W were more virulent than those of serogroup Y [31].

The C and the W isolates in our study belonged to the ST-32 clonal complex and ST-11 clonal complex respectively, which are both considered to be hyperinvasive [8]. Meanwhile, the Y isolates belonged to the ST-23 clonal complex, and the B isolates, though mainly of the hyperinvasive ST-41/44 clonal complex, showed weaker capsule expression based on the co-agglutination results, with several isolates marked as negative or +/-. These factors should all be taken into consideration when interpreting the results, since they might at least partly explain the outcome. Notably, these highly invasive isolates were of different *porB* classes; the W isolates were of *porB* class 2 and the C isolates were of *porB* class 3, and, as mentioned, no association was found between highly virulent isolates and *porB* class.

The results from the experiments with the *porB* mutants suggest that the *porB* gene itself plays a critical role for infection, as the mice infected with the mutants that had inactivated *porB* were less ill than the mice infected with the parental invasive isolates. This was reflected by higher clinical scores, significantly lower bacteraemia, and a lower inflammatory response. PorB is an outer membrane porin which plays a role in microbial virulence. The protein can insert itself into mitochondrial membranes, which induces cell apoptosis and enhances invasiveness through induction of Ca2 + influx and activation of toll-like receptors [2, 16]. This may explain why the mutated meningococci without the *porB* gene showed less virulence.

The invasive isolate 19–977 that was mutated was of *porB* class 3, and the carriage isolate Mcbar-21 was of *porB* class 2. It is still unclear whether the *porB* class itself is important for meningococcal virulence, and not just the presence of the gene. In the GWAS, *porB* class 3 was linked with carriage isolates [15]. Interestingly, in the previously mentioned mouse trial comparing W and Y strains, the W isolates were all of *porB* class 2 [31]. Furthermore, previous studies with transgenic rats have shown that isolates expressing *porB* class 2 exhibit higher virulence than those expressing *porB* class 3. This suggests that *porB* class 2 may enhance immune evasion capabilities [32, 33].

One study investigated strains of meningococci that were engineered to lack certain molecules known to inhibit the alternative pathway of the immune system, and found that some strains still managed to inhibit the alternative pathway. These strains expressed PorB class 2 protein, which appeared to help the bacteria resist the immune response by binding certain parts of the human complement Factor H and by reducing the deposition of the immune protein complement component 3 (C3). This was linked to resistance to the body’s complement system, which is a key part of the immune defence against infections. This resistance mechanism was specific to humans, as animal experiments showed that the bacteria did not resist the immune system in rabbits or mice [32]. These findings could potentially explain why our study could not find stronger differences regarding *porB* class and outcome of infection in mice. Further investigations are needed to fully address the role of *porB* class.

A limitation of this study is that relatively few isolates and mice were used. Experiments with more isolates and more mice could generate even more significant data, although this must be considered in relation to the ethical imperative to use as few mice as possible while still yielding reliable results. Another limitation is the use of a mouse model. Although it is possible to sustain an infection with *N. meningitidis* in these mice, since they have been modified to express human transferrin, it is important to remember that the meningococcus is a human pathogen. The bacteria might act somewhat differently in mice, as observed in the abovementioned study regarding *porB* class, which might explain why we did not find a clearer link between *porB* class and outcome of infection. A third limitation of the study is that only a small number of successful *porB* mutants with inactivated *porB* were used. The mutation process was challenging, and thus only two isolates were successfully mutated. Ideally, more isolates would have been mutated through *porB* inactivation. To increase the number of mutations in future studies, the transformation procedure could be modified– for example, by replacing natural transformation with electroporation or conjugation, which may help circumvent restriction barriers related to DNA uptake. Additionally, creating mutations in other outer membrane proteins would provide a broader context for comparison and could help describe the specific role of PorB.

In another study using GWAS on *N. meningitidis*, several genetic variants, including SNPs, were associated with meningococci of carriage or invasive type. These genetic variants included a significant association of invasive isolates with polymorphisms in the *fHbp* gene. Notably, the strongest associations were found in genes involved in producing meningococcal surface structures that interact with host cells and immune molecules, as well as in genes linked to their expression [34]. In another study examining invasive strains in New Zealand, carriage strains from household contacts were analysed simultaneously. Although the isolates were very similar according to laboratory typing methods, they did have genetic differences. Several carriage strains had also lost their type IV pili, and this was correlated with reduced TNF-α expression when cultured with epithelial cells [35]. A study of an epidemic of meningococcal outbreak in West Africa revealed that invasive disease had resulted from a previously asymptomatic carriage strain that had acquired virulence genes, including capsule genes [36]. These findings are in line with our results, and support the notion that genetic factors, in addition to capsule expression, play a major role in disease development.

Other genes found to be associated with the carriage isolates in our previous GWAS [15] were *tspB*, encoding for T and B cell-stimulating protein B, which mediates the binding of human IgG and the formation of biofilm and is also involved in the pathogenesis of *N. meningitidis* [37], and the *PilE/S genes*, encoding a subunit of the pili. Two SNPs were also found to be associated with invasiveness or carriage; one SNP in the gene *glmU*, which encodes for an enzyme involved in synthesis of a substrate involved in the synthesis of lipooligosaccharide and the polysaccharide capsule, and one SNP in the gene *fkpB*, which encodes an enzyme involved in protein folding. The role of these genetic variants in promoting invasive infection or severity of infection still remains to be explored phenotypically in functional experiments.

## Conclusion

This study confirms that in comparison to carriage isolates, invasive isolates of *N. meningitidis* have a greater ability to cause an invasive infection in transgenic mice, which emphasizes the capacity of the isolate itself to cause infection. However, this increased ability was most apparent among serogroup C and W isolates, while serogroup B isolates (both carriage and invasive) were overall of lower virulence and not significantly different from each other. The results highlight the importance of the intrinsic properties and the genetic signature of the bacteria, including the impact of the *porB* gene, on meningococcal virulence. Variants of the *porB* gene could also to some extent be linked to phenotypic outcome. However, since *porB* class alone did not consistently explain differences in virulence, its contribution may depend on the broader genomic context of the isolate. Our data underline that in addition to host and environmental factors, bacterial factors are key determinants in the outcome of *N. meningitidis*-host interaction.

## Supplementary Information

Supplementary Material 1.

Supplementary Material 2.

Supplementary Material 3.

## Data Availability

All of the datasets from the animal experiments are openly available in FigShare at http://doi.org/10.6084/m9.figshare.28239164. Sequence data of the isolates are publicly available on the PubMLST Neisseria database.

## Abbreviations

cc: Clonal Complex
CFU: Colony forming unit
cnl: Capsule Null Locus
CXCL1/KC: CXC Chemokine Ligand 1/Keratinocyte-Derived Cytokine
GWAS: Genome-Wide Association Study
IL-6: Interleukin-6
IMD: Invasive Meningococcal Disease
PorA: Porin A
PorB: Porin B
SNPs: Single Nucleotide Polymorphisms
ST: Sequence Type
TNF-α: Tumour Necrosis Factor Alpha

## References

[1] Rosenstein NE, Perkins BA, Stephens DS, Popovic T, Hughes JM. Meningococcal disease. N Engl J Med. 2001;344:1378–88. 11333996 10.1056/NEJM200105033441807

[2] Rouphael NG, Stephens DS. *Neisseria meningitidis*: biology, microbiology, and epidemiology. Methods Mol Biol. 2012;799:1–20. 21993636 10.1007/978-1-61779-346-2_1 PMC4349422

[3] Parikh SR, Campbell H, Bettinger JA, Harrison LH, Marshall HS, Martinon-Torres F, et al. The everchanging epidemiology of meningococcal disease worldwide and the potential for prevention through vaccination. J Infect. 2020;81:483–98. 32504737 10.1016/j.jinf.2020.05.079

[4] Voss SS, Nielsen J, Valentiner-Branth P. Risk of sequelae after invasive meningococcal disease. BMC Infect Dis. 2022;22:148. 35148717 10.1186/s12879-022-07129-4 PMC8831877

[5] Caugant DA, Maiden MCJ. Meningococcal carriage and disease—population biology and evolution. Vaccine. 2009;27:B64–70. 19464092 10.1016/j.vaccine.2009.04.061 PMC2719693

[6] Watle SV, Caugant DA, Tunheim G, Bekkevold T, Laake I, Brynildsrud OB, et al. Meningococcal carriage in Norwegian teenagers: strain characterisation and assessment of risk factors. Epidemiol Infect. 2020;148:e80. 32228726 10.1017/S0950268820000734 PMC7189347

[7] Maiden MC, Bygraves JA, Feil E, Morelli G, Russell JE, Urwin R, et al. Multilocus sequence typing: a portable approach to the identification of clones within populations of pathogenic microorganisms. Proc Natl Acad Sci U S A. 1998;95:3140–5. 9501229 10.1073/pnas.95.6.3140 PMC19708

[8] Zarantonelli ML, Lancellotti M, Deghmane AE, Giorgini D, Hong E, Ruckly C, et al. Hyperinvasive genotypes of *Neisseria meningitidis* in France. Clin Microbiol Infect. 2008;14:467–72. 18294240 10.1111/j.1469-0691.2008.01955.x

[9] Caugant DA. Genetics and evolution of *Neisseria meningitidis*: importance for the epidemiology of meningococcal disease. Infect Genet Evol. 2008;8:558–65. 18479979 10.1016/j.meegid.2008.04.002

[10] Jusot J-F, Neill DR, Waters EM, Bangert M, Collins M, Moreno LB, et al. Airborne dust and high temperatures are risk factors for invasive bacterial disease. J Allergy Clin Immunol. 2017;139:977. 27523432 10.1016/j.jaci.2016.04.062 PMC5338876

[11] Dwilow R, Fanella S. Invasive meningococcal disease in the 21st century—an update for the clinician. Curr Neurol Neurosci Rep. 2015;15:2. 25637287 10.1007/s11910-015-0524-6

[12] Taha M-K, Weil-Olivier C, Bouée S, Emery C, Nachbaur G, Pribil C, et al. Risk factors for invasive meningococcal disease: a retrospective analysis of the French National public health insurance database. Hum Vaccines Immunother. 2021;17:1858–66. 10.1080/21645515.2020.1849518 PMC8115611 33449835

[13] Stephens DS. Biology and pathogenesis of the evolutionarily successful, obligate human bacterium *Neisseria meningitidis*. Vaccine. 2009;27:B71-7. 19477055 10.1016/j.vaccine.2009.04.070 PMC2712446

[14] Tzeng Y-L, Thomas J, Stephens DS. Regulation of capsule in neisseria meningitidis. Crit Rev Microbiol. 2016;42:759–72. 26089023 10.3109/1040841X.2015.1022507 PMC4893341

[15] Eriksson L, Johannesen TB, Stenmark B, Jacobsson S, Säll O, Hedberg ST, et al. Genetic variants linked to the phenotypic outcome of invasive disease and carriage of neisseria meningitidis. Microb Genomics. 2023;9:001124. 10.1099/mgen.0.001124 PMC10634450 37874326

[16] Tanabe M, Nimigean CM, Iverson TM. Structural basis for solute transport, nucleotide regulation, and immunological recognition of *Neisseria meningitidis* PorB. Proc Natl Acad Sci U S A. 2010;107:6811–6. 20351243 10.1073/pnas.0912115107 PMC2872391

[17] Smith NH, Maynard Smith J, Spratt BG. Sequence evolution of the PorB gene of *Neisseria gonorrhoeae* and *Neisseria meningitidis*: evidence of positive darwinian selection. Mol Biol Evol. 1995;12:363–70. 7739379 10.1093/oxfordjournals.molbev.a040212

[18] Zarantonelli M-L, Szatanik M, Giorgini D, Hong E, Huerre M, Guillou F, et al. Transgenic mice expressing human transferrin as a model for meningococcal infection. Infect Immun. 2007;75:5609–14. 17893132 10.1128/IAI.00781-07 PMC2168318

[19] Szatanik M, Hong E, Ruckly C, Ledroit M, Giorgini D, Jopek K, et al. Experimental meningococcal sepsis in congenic transgenic mice expressing human transferrin. PLoS One. 2011;6:e22210. 21811575 10.1371/journal.pone.0022210 PMC3141004

[20] Olof S, Lorraine E, Asfaw Idosa B, Alexander P, Anders M, Sara TH, et al. Prevalence and persistence of neisseria meningitidis carriage in Swedish university students. Epidemiol Infect. 2023;151:e25. 36775828 10.1017/S0950268823000018 PMC9990396

[21] Kellogg DS, Peacock WL, Deacon WE, Brown L, Pirkle DI. *Neisseria gonorrhoeae*. i. Virulence genetically linked to clonal variation. J Bacteriol. 1963;85:1274–9. 14047217 10.1128/jb.85.6.1274-1279.1963 PMC278328

[22] Deghmane A-E, Veckerlé C, Giorgini D, Hong E, Ruckly C, Taha M-K. Differential modulation of TNF-α–induced apoptosis by *Neisseria meningitidis*. PLoS Pathog. 2009;5:e1000405. 19412525 10.1371/journal.ppat.1000405 PMC2669886

[23] Levy M, Antunes A, Fiette L, Deghmane A-E, Taha M-K. Impact of corticosteroids on experimental meningococcal sepsis in mice. Steroids. 2015;101:96–102. 26066898 10.1016/j.steroids.2015.05.013

[24] American Veterinary Medical Association, AVMA. AVMA guidelines for the euthanasia of animals: 2020 edition. Schaumburg, IL: AVMA; 2020.

[25] Tanaka T, Narazaki M, Kishimoto T. IL-6 in inflammation, immunity, and disease. Cold Spring Harb Perspect Biol. 2014;6: a016295. 25190079 10.1101/cshperspect.a016295 PMC4176007

[26] Parameswaran N, Patial S. Tumor necrosis factor-α signaling in macrophages. Crit Rev Eukaryot Gene Expr. 2010;20:87–103. 21133840 10.1615/critreveukargeneexpr.v20.i2.10 PMC3066460

[27] Pathan N. Pathophysiology of meningococcal meningitis and septicaemia. Arch Dis Child. 2003;88:601–7. 12818907 10.1136/adc.88.7.601 PMC1763171

[28] Waage A, Brandtzaeg P, Halstensen A, Kierulf P, Espevik T. The complex pattern of cytokines in serum from patients with meningococcal septic shock. Association between interleukin 6, interleukin 1, and fatal outcome. J Exp Med. 1989;169:333–8. 2783334 10.1084/jem.169.1.333 PMC2189201

[29] Moser B, Clark-Lewis I, Zwahlen R, Baggiolini M. Neutrophil-activating properties of the melanoma growth-stimulatory activity. J Exp Med. 1990;171:1797–802. 2185333 10.1084/jem.171.5.1797 PMC2187876

[30] Sawant KV, Poluri KM, Dutta AK, Sepuru KM, Troshkina A, Garofalo RP, et al. Chemokine CXCL1 mediated neutrophil recruitment: role of glycosaminoglycan interactions. Sci Rep. 2016;6:33123. 27625115 10.1038/srep33123 PMC5021969

[31] Eriksson L, Stenmark B, Deghmane A-E, Thulin Hedberg S, Säll O, Fredlund H, et al. Difference in virulence between *Neisseria meningitidis* serogroups W and Y in transgenic mice. BMC Microbiol. 2020;20:92. 32295520 10.1186/s12866-020-01760-4 PMC7160935

[32] Lewis LA, Vu DM, Vasudhev S, Shaughnessy J, Granoff DM, Ram S. Factor H-dependent alternative pathway inhibition mediated by porin B contributes to virulence of *Neisseria meningitidis*. mBio. 2013;4:e00339-13. 24129254 10.1128/mBio.00339-13 PMC3812710

[33] Lewis LA, Vu DM, Granoff DM, Ram S. Inhibition of the alternative pathway of nonhuman infant complement by Porin B2 contributes to virulence of *Neisseria meningitidis* in the infant rat model. Infect Immun. 2014;82:2574–84. 24686052 10.1128/IAI.01517-14 PMC4019150

[34] Earle SG, Lobanovska M, Lavender H, Tang C, Exley RM, Ramos-Sevillano E, et al. Genome-wide association studies reveal the role of polymorphisms affecting factor H binding protein expression in host invasion by *Neisseria meningitidis*. PLoS Pathog. 2021;17:e1009992. 34662348 10.1371/journal.ppat.1009992 PMC8553145

[35] Ren X, Eccles DA, Greig GA, Clapham J, Wheeler NE, Lindgreen S, et al. Genomic, transcriptomic, and phenotypic analyses of neisseria meningitidis isolates from disease patients and their household contacts. mSystems. 2017;2. 10.1128/msystems.00127.17. 10.1128/mSystems.00127-17 PMC5686521 29152586

[36] Brynildsrud OB, Eldholm V, Bohlin J, Uadiale K, Obaro S, Caugant DA. Acquisition of virulence genes by a carrier strain gave rise to the ongoing epidemics of meningococcal disease in West Africa. Proc Natl Acad Sci U S A. 2018;115:5510–5. 29735685 10.1073/pnas.1802298115 PMC6003489

[37] Müller MG, Ing JY, Cheng MK-W, Flitter BA, Moe GR. Identification of a phage-encoded Ig-binding protein from invasive *Neisseria meningitidis*. J Immunol Baltim Md 1950. 2013;191:3287–96. 10.4049/jimmunol.1301153 PMC3780609 23926326

[38] Percie du Sert N, Hurst V, Ahluwalia A, Alam S, Avey MT, Baker M, et al. The ARRIVE guidelines 2.0: updated guidelines for reporting animal research. PLoS Biol. 2020;18:e3000410. 32663219 10.1371/journal.pbio.3000410 PMC7360023

[39] PubMLST. Neisseria spp. https://pubmlst.org/organisms/neisseria-spp (accessed 5 Nov 2024).

