# Supplementary figures and images for: Inactivation of the porB gene reduces the virulence of Neisseria meningitidis in transgenic mice
Source: BMC Microbiol. 2025 Aug 16;25:515. doi: 10.1186/s12866-025-04246-3 (PMC12357335; doi:10.1186/s12866-025-04246-3)

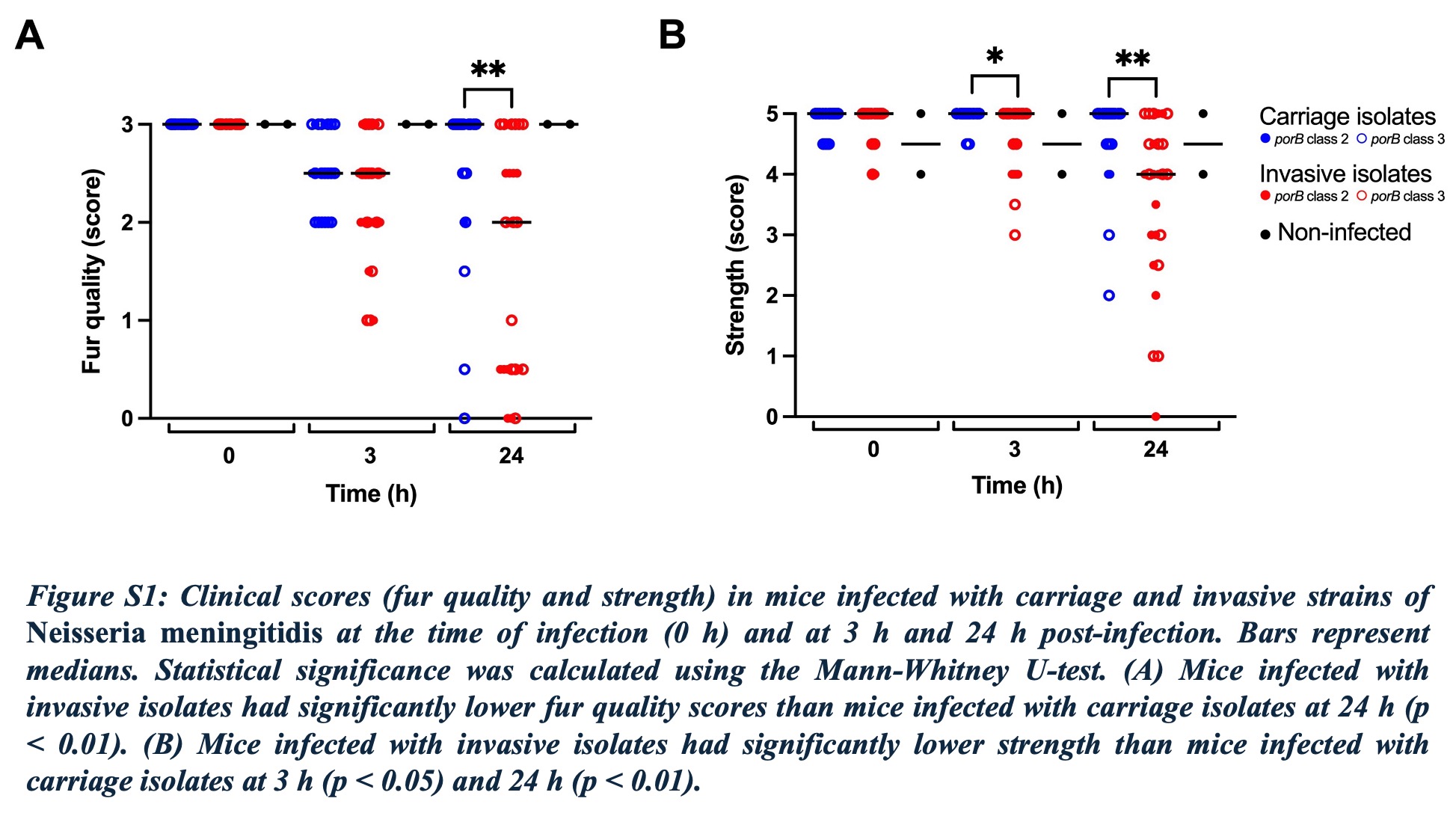

Supplement: Supplementary file 2 — Supplementary Material 2. [file 12866_2025_4246_MOESM2_ESM.jpg]

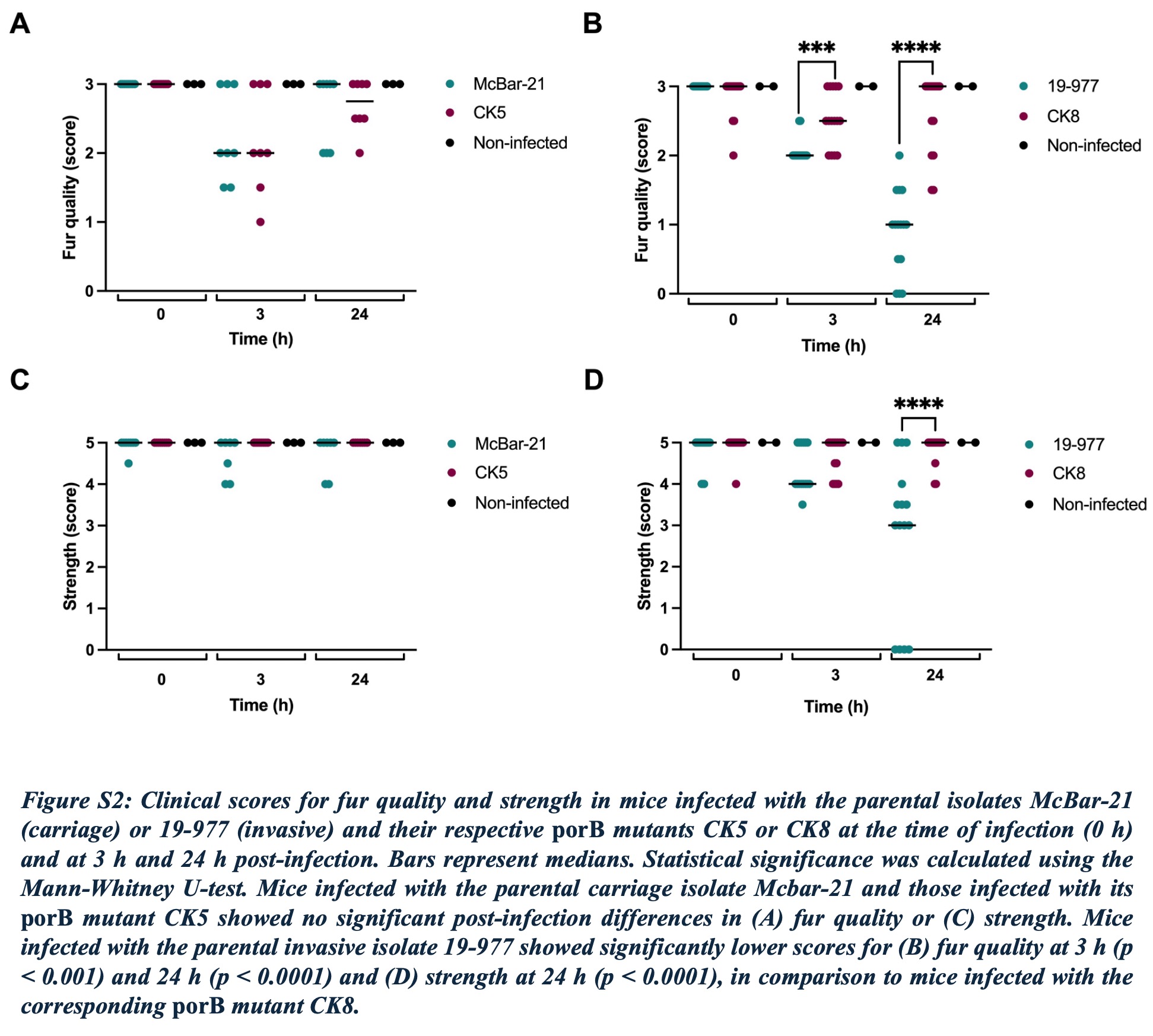

Supplement: Supplementary file 3 — Supplementary Material 3. [file 12866_2025_4246_MOESM3_ESM.jpg]
